# 911. Impact of Intensified Clindamycin Stewardship Initiatives in Three Phases: A Quasi-Experimental Study

**DOI:** 10.1093/ofid/ofac492.756

**Published:** 2022-12-15

**Authors:** Kelly C Gamble, Dusten T T Rose, Kristin Mondy, Rama Thyagarajan, Theresa Jaso, Leslie Coats, Kelly R Reveles

**Affiliations:** McLeod Regional Medical Center; Dell Seton Medical Center at the University of Texas, Austin, Texas; Dell Seton Medical Center at the University of Texas, Dell Medical School at the University of Texas Austin, Austin, Texas; Dell Seton Medical Center at the University of Texas, Dell Medical School at the University of Texas Austin, Austin, Texas; Ascension Seton Medical Center Austin, Austin, Texas; University of Texas at Austin College of Pharmacy, Austin, Texas; The University of Texas at Austin, San Antonio, Texas

## Abstract

**Background:**

Clindamycin utilization has provoked gram-positive and anaerobic bacterial resistance and is associated with *Clostridioides difficile* infections (CDI). The purpose of this study was to evaluate if implementing local clindamycin-focused stewardship initiatives impacts clindamycin utilization and outcome metrics.

**Methods:**

This multicenter, retrospective quasi-experimental study was conducted in adult hospitalized patients who received clindamycin from 2018 to 2020. Outcomes were compared in three phases between an intervention and control group (Figure 1). The primary outcome was inappropriate utilization: a resistant pathogen, concurrent antibiotic(s) with pathogen activity, a non-necrotizing infection, an alternative agent was preferred, or alternative reasons per expert discretion. Secondary outcomes included clindamycin duration of therapy per 1000 patient hospital days (DOT/1000 PD), 30-day CDI, 30-day readmission, and in-hospital mortality. Outcomes were compared between groups using the chi-square, Fisher’s exact, or Kruskal-Wallis tests as appropriate.

**Figure 1.**
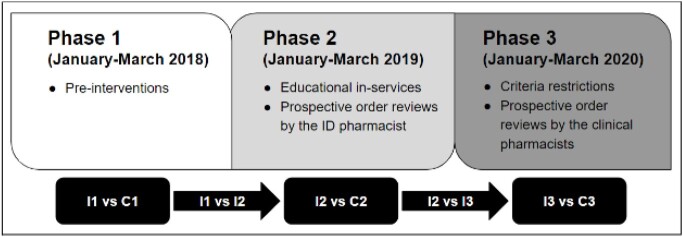
Study Design

**Results:**

The study included 481 patients (Table 1). Inappropriate clindamycin use in the intervention group was highest in Phase 1 (94%), then decreased in Phase 2 (72%) and Phase 3 (74%) (p< 0.01). Comparing Phase 1, Phase 2, and Phase 3 between the intervention and control groups, respectively, the primary outcome occurred in 94% vs 93% (p=0.80), 72% vs 88% (p=0.02), and 74% vs 89% (p=0.03). The DOT/1000 PD was 10 in Phase 1, 9.2 in Phase 2, and 6.2 in Phase 3. Initiatives were not associated with significant reductions in 30-day CDI (1% in Phase 1, 0% in Phase 2, 0% in Phase 3, p=0.64), 30-day readmission (18% in Phase 1, 31% in Phase 2, 22% in Phase 3, p=0.13), or in-hospital mortality (0% in Phase 1, 2% in Phase 2, 0% in Phase 3, p=0.33).

**Table 1.**
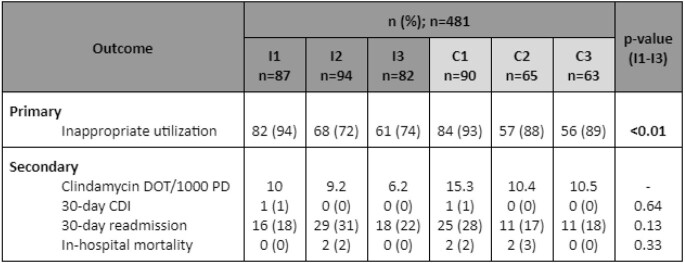
Outcomes

**Conclusion:**

Clindamycin-focused stewardship initiatives reduced inappropriate prescribing patterns by approximately 20% but still remained high in Phase 3. Clindamycin was primarily ordered in the emergency department (ED). Additional initiatives may include formal criteria for use and removing clindamycin from automated dispensing cabinets in the ED. Our initiatives may serve as a model for other institutions to reduce clindamycin utilization and its associated complications.

**Disclosures:**

**All Authors**: No reported disclosures.

**Figure 1.** Study Design

ID: infectious diseases, I: intervention group, C: control group

**Table 1.** Outcomes

I: intervention group, C: control group, DOT/1000 PD: days of therapy per 1000 patient days, CDI: Clostridioides difficile infection

